# The interrelationship between sleep disturbance symptoms and aggression before and after the campus closure of the COVID-19 pandemic: insight from a cross-lagged panel network model

**DOI:** 10.3389/fpubh.2024.1357018

**Published:** 2024-03-21

**Authors:** Jinhua Zou, Baohua Bian, Min Li, Gang Liu

**Affiliations:** ^1^Lianyungang Fourth People's Hospital, Lianyungang, China; ^2^Department of Psychiatry, Affiliated Nanjing Brain Hospital, Nanjing Medical University, Nanjing, China

**Keywords:** sleep disturbance, symptoms, aggression, cross-lagged panel network, college students

## Abstract

**Background:**

The COVID-19 pandemic is detrimental to sleep quality and increases aggression among college students. Nevertheless, relevant studies were rare. Hence, we collected longitudinal data during and post-campus closure in the current study to investigate the relationship between sleep disturbance and aggression.

**Methods:**

Data from 665 college students (59.2% females, *Mean*_age_ = 19.01, SD _age_ = 1.25) were collected before (wave 1) and after (wave 2) the campus closure of COVID-19. All participants were asked to fill out the Buss-Perry Aggression Questionnaire and the Youth Self-Rating Insomnia Scale. Two symptom networks and a cross-lagged panel network were formed and tested.

**Results:**

Hostility has the highest centrality in the symptom network both in waves 1 and 2, and it bridges sleep disturbance and aggression. “Easily be woken” – “wake up too early” and “wake up with tired” – “function hindrance” are two important symptom associations in networks of waves 1 and 2. All symptoms except “*difficulty in falling asleep*” and “*easily be woken*” ameliorated after closure. Moreover, “*physical aggression*” and “hostility” can trigger other symptoms in wave 2.

**Conclusion:**

As the first study about aggression and sleep disturbance in the background of COVID-19, we provide valuable information about the relationship between sleep disturbance and aggression on the symptom dimension.

## 1 Introduction

Due to the COVID-19 pandemic, college students have witnessed and experienced school closures or the transition to online teaching (1). While strict containment measures aim to prevent the spread of the virus, they also have wide-ranging effects on students’ wellbeing (2). Deng et al. (3) found that 33% of college students suffer from sleep disturbances during COVID-19, particularly during online courses (4–7).

Poor sleep quality among college students is associated with an increased likelihood of aggression (8). This is because sleep deprivation affects prefrontal cortical functioning, leading to a loss of emotional control and ultimately triggering aggressive behavior (9). In the context of COVID-19, sleep disturbance is also closely linked to interpersonal violence (10) and aggression (11). However, the relationship between sleep disturbance and aggression during and after campus closure has been rarely studied. To address this gap, the current study collected longitudinal data and investigated the relationship between sleep disturbance and aggression in a sample of college students affected by the COVID-19 campus closures.

From a psychological perspective, aggression is a typical behavior characterized by purposeful attacks or hostility toward others (12). The general aggression model (GAM) (13) offers a comprehensive theoretical framework that integrates both personal factors (e.g., cognitions, feelings, and emotional arousal) and situational factors (such as COVID-19), which ultimately influence behaviors such as aggression. A meta-analysis revealed that lockdown characteristics, such as isolation, restricted social contact, quarantine duration, and limitations, significantly increase college students’ negative emotional symptoms, including anxiety, depression, and stress (14). Moreover, longitudinal studies found that negative emotional arousal resulting from sudden public health events may trigger adolescent aggression (15, 16). Research on college students indicates a close association between sleep disturbance and anxiety and depression during COVID-19, which can serve as risk factors for aggressive decision-making (5, 17–20).

In the proximal path perspective of the GAM (13), contingent situations, such as campus closures, can significantly impact aggressive decision-making. Specifically, Mazza et al. (21) and Overall et al. (22) noted a significant increase in aggression during campus closures. Birmingham et al. (23) indicated that college students experienced restless sleep during the lockdown period, which could trigger aggression. Additionally, in a survey conducted by Kormukcu (24), college students exhibited more anger during university closures, although overall aggression did not increase significantly. Given the inconsistent results mentioned above, further study to explore the relationship between sleep disturbance and aggression during the lockdown period is of great necessity.

Nevertheless, solely considering the proximal path is insufficient to fully explain the mechanism linking sleep disturbance and aggression. The distal path perspective of the GAM should also be considered (13). In the distal path, long-lasting environmental modifiers or biological factors, such as peer relationships, family background, and testosterone levels, influence personal traits related to aggression arousal (25–27). For college students, COVID-19 has disrupted their educational routine by shifting from offline teaching to online teaching, with uncertainty regarding when life will return to pre-pandemic norms (28). According to the social displacement hypothesis (29), college students may spend more time engaging in online courses, using social media, or playing video games to cope with negative emotions, thereby increasing sleep disturbances and aggression (30, 31). Additionally, considering biological factors in the distal path, sleep disturbance can disrupt testosterone rhythms, which are closely linked to aggressive behavior (32, 33).

The COVID-19 pandemic has brought about sleep disturbances for college students who are already contending with significant academic stress, changes in course delivery, and diminished motivation (34). College students often rely on media to maintain interpersonal relationships, but the side effect of problematic media use is poor sleep quality, which can contribute to aggressive behavior (35). Additionally, chronic exposure to social media and competitive video games has been associated with the provocation of aggressive behavior (36, 37). In such aggressive environments, college students’ sleep is put at risk (38). Furthermore, individuals with severe aggressive tendencies show abnormal theta and delta power during the sleep stage (39). Considering the bidirectional relationship between sleep disturbance and aggression and recognizing the negative effects of both on mental and physical health, untangling their relationship in the context of COVID-19 is of significant importance.

However, in almost all previous studies, aggression or sleep disturbance has been considered a latent variable (34, 40, 41). Aggression and sleep disturbance encompass a dynamic cluster of dimensions and symptoms that interact dynamically to manifest the variable (42–44). For instance, as illustrated by Borsboom and Cramer (45), a college student who wakes up too early due to nightmares may exhibit hostility toward peers online. Consequently, feeling guilty, the college student may experience difficulties falling asleep in the evening. To uncover the bidirectional relationship between sleep disturbance and aggression on the symptom dimension, the network approach is optimal, as it renders symptoms (nodes) and associations between symptoms (edges) visible (46). Furthermore, through network analysis, key symptoms linking aggression and sleep disturbance can be readily identified (47). Additionally, by employing a cross-lagged panel network (CLPN), we can even identify symptoms during the COVID-19 campus closures that may trigger other symptoms after the closures.

In the existing research, Li et al. (48) and Hirota et al. (49) formed and analyzed the network structure of aggression in patients with schizophrenia and autism. However, to our knowledge, no study has been conducted with network analysis on the association between sleep disturbance and aggression among college students. As previously mentioned, since COVID-19 has introduced new norms to college life (50), in the current study, based on network analysis, we aimed to start from symptoms to depict a vivid map of college life from the perspective of aggression and sleep disturbance.

In summary, in the current study, we have five goals: (1) to find critical symptoms or behaviors in sleep disturbance and aggression; (2) to identify essential associations from the perspective of sleep disturbance and aggression; (3) to portray bridge symptoms that can connect sleep disturbance and aggression; (4) to identify how levels of symptoms will change with the lifting of closures through longitudinal data; and (5) to depict key symptoms before (wave 1) that can cause other symptoms after (wave 2) the campus closure due to COVID-19.

## 2 Method

### 2.1 Participants and procedure

The baseline data met the criterion that all participants had not experienced campus quarantine. Subsequently, all participants met the criteria of residing on campus and experiencing at least 1 month of mandatory school lockdown, during which students were not allowed to leave the school premises and could only engage in activities and studies on campus. One month after the school lifted the lockdown, college students were allowed to freely enter and exit the campus, and the second round of data collection was conducted. Hence, the baseline datasets (October to November 2021) contained 1,302 participants (*Mean*_age_ = 19.38, SD_age_ = 1.32; N _female_ = 847), while the second wave of datasets (January 2022) included 1,359 participants (*Mean*_age_ = 19.65, SD _age_ = 1.45; N _female_ = 815), both from a university in China. When the datasets from both waves were combined according to the student’s school numbers, 665 students (*Mean*_age_ = 19, SD _age_ = 1.25; N _female_ = 394) were included in the final analysis. All participants ultimately passed a validation question in the electronic questionnaire, which presented the prompt: “Among the four options, lion, dog, cat, and panda, please select the panda.”

All datasets were collected via the online questionnaire platform “Wenjuanxing”.^1^ Students were asked to provide signed informed consent before participation. The research was examined and approved by the Ethics Committee of the First Author’s Affiliated Institution.

### 2.2 Measures

#### 2.2.1 Buss-Perry aggression questionnaire

The 29-item Buss-Perry Aggression Questionnaire (BPAQ) assesses the tendency toward aggression (42). Each item is rated from 1 (very unlike) to 5 (very like). The BPAQ has four empirical subscales: physical aggression (nine items), verbal aggression (five items), anger (seven items), and hostility (eight items). Physical aggression and verbal aggression are both motor components of behavior, while anger is the emotional or affective component of aggressive behavior, and hostility is the cognitive component of behavior. The Chinese version was revised by Li et al. (51). In the present study, the Cronbach *α* values of four factors were 0.80, 0.65, 0.75, and 0.84, respectively.

#### 2.2.2 Youth self-rating insomnia scale

The Youth Self-Rating Insomnia Scale (YSIS) is a 5-point Likert questionnaire assessing sleep disturbance in the past month (43). Participants answered two questions about overall sleep quality and six about the frequency of specific sleep disturbance symptoms. Total scores ranged from 8 to 40, and higher scores indicated poorer sleep quality. The Chinese version was revised by Liu et al. (52). In the current study, YSIS has a high internal consistency with the Cronbach *α* value of 0.91.

### 2.3 Network analysis

#### 2.3.1 Item check

We used *R* version 4.2.1 (53) to perform all analyses. *Means*, *standard deviation* (SD), *skewness*, and *kurtosis* of all item scores were calculated. We assessed item redundancy using the *R* package *networktools 1.5.0* (54). For informativeness, items should be excluded if their scores were 1.5 SD below the mean item SD (i.e., poorly informative). For redundancy, if more than 75% of correlations between two variables and all other variables were not significantly different, these two variables were considered redundant.

#### 2.3.2 Cross-sectional network estimation

An extended Bayesian information criterion (EBIC) graphical least absolute shrinkage and selection operator (LASSO) model (55) was used to estimate the network. Each node (i.e., item) in the network represents a symptom, and each edge represents the partial correlation between two symptoms. The correlation matrix was shrunk to obtain simpler and sparser networks. Blue and red edges denote positive and negative correlations, respectively. The R packages *bootnet 1.4.3* and *qgraph 1.6.9* were employed for network estimation and visualization (46, 56). The expected influence (*EI*) was used to assess the centrality of nodes in this study. Predictability (i.e., *R*^2^) was estimated using the *R* package *mgm 1.2–12* (57). Bridge symptoms serve as the channel connection between different disorders (47). Following previous research (58, 59), we screened bridge symptoms based on the criterion of standardized values of bridge strength ≥1 in the current study.

#### 2.3.3 Cross-lagged network estimation

A CLPN was conducted to examine the connections between the first and second assessments over time by using the *glmnet* package (60). A CLPN illustrates how a single node (i.e., symptom) at the first time point predicts other nodes at the second time point after adjusting for all other variables at the first time point. The directed edges of each node pointing to itself represent the autoregressive coefficients, while the directed edges pointing to other nodes represent the cross-lagged coefficients. The color of the arrows indicates the directionality of the effect, with green arrows indicating positive effects and red arrows indicating negative effects. The line thickness indicates the strength of the association. To simplify the network, we employed the LASSO approach to shrink small regression coefficients to 0. For directed CLPNs, we calculated two centrality indices: cross-lagged “in expected influence” (*IEI*) and “out expected influence” (*OEI*). *IEI* signifies the degree to which one symptom is predicted by other symptoms (i.e., the sum of values of incoming edges associated with one symptom), while *OEI* signifies the degree to which one symptom can predict other symptoms (i.e., the sum of values of outgoing edges associated with one symptom).

#### 2.3.4 Network comparison

The network comparison test (NCT) was employed to evaluate the difference between edge invariance (distributions of edge weights) and global strength (the sum of all edge weights) between the networks in waves 1 and 2 using the *R* package *NetworkComparisonTest 2.2.1* (61).

#### 2.3.5 Network stability and accuracy

The case-dropping bootstrap procedure was used to assess the stability of centrality indices (46), providing the correlation stability coefficient (*CS-C*). The *CS-C* represented the proportion of samples that could be removed, with a 95% probability that the correlation between the original centrality indices would be at least 0.70. Generally, the *CS-C* should be ≥0.25, preferably ≥0.50. Bootstrapped confidence intervals (95% *CI*s) were computed to analyze the accuracy of edges. Narrower *CI*s indicated a more accurate network. Differences between edge weights and centrality strengths were also analyzed by bootstrap tests based on 0.95 *CIs*. If *CIs* did not include zero, there was a statistical difference between two edges or two nodes. All analyses were performed using the *R* package *bootnet 1.4.3* (46).

## 3 Results

### 3.1 Descriptive statistics and item check

The item check results revealed that YSIS1-YSIS5 and YSIS1-YSIS2 are redundant. Specifically, only 20% of correlations were significantly different for YSIS1 and YSIS5, and only 16.7% of correlations were significantly different for YSIS1 and YSIS2. Considering that YSIS1 and YSIS2 pertain to overall sleep quality rather than specific symptoms, we excluded them from the subsequent analyses. No items were found to be poorly informative. The *means*, *SDs*, *skewness*, *kurtosis,* and *t*-test results of all symptoms are shown in Table 1. There were no significant differences in gender across all variables in both waves 1 and 2 (*p* > 0.05).

**Table 1:** Descriptive information and *t*-test results of data from two-time points.

	First wave				Second wave				*t*-test	
	Mean	SD	Skew	Kurtosis	Mean	SD	Skew	Kurtosis	*p*	Cohen’s d
BPAQ1	1.99	0.69	0.57	0.17	1.9	0.64	0.47	−0.6	−0.09	−0.14
BPAQ2	2.64	0.67	−0.15	0.65	2.46	0.74	−0.32	0.01	−0.18	−0.24
BPAQ3	2.3	0.71	0.2	−0.33	2.19	0.67	0.29	−0.32	−0.10	−0.15
BPAQ4	2.38	0.75	0	−0.27	2.13	0.77	0.14	−0.69	−0.24	−0.33
YSIS3	1.94	1.07	0.9	0.04	1.96	1.07	0.86	−0.09	0.03	0.02
YSIS4	1.77	1.05	1.24	0.76	1.68	0.95	1.25	0.79	−0.09	−0.08
YSIS5	1.73	1.04	1.31	0.85	1.58	0.92	1.59	1.93	−0.15	−0.13
YSIS6	2.2	1.22	0.67	−0.61	1.72	1.01	1.31	0.9	−0.48	−0.36
YSIS7	2.36	1.27	0.58	−0.69	1.87	1.08	1.08	0.31	−0.49	−0.36
YSIS8	1.88	1.09	1.1	0.42	1.64	0.95	1.47	1.46	0.25	−0.21

### 3.2 Cross-sectional symptom networks

**Figure 1:**
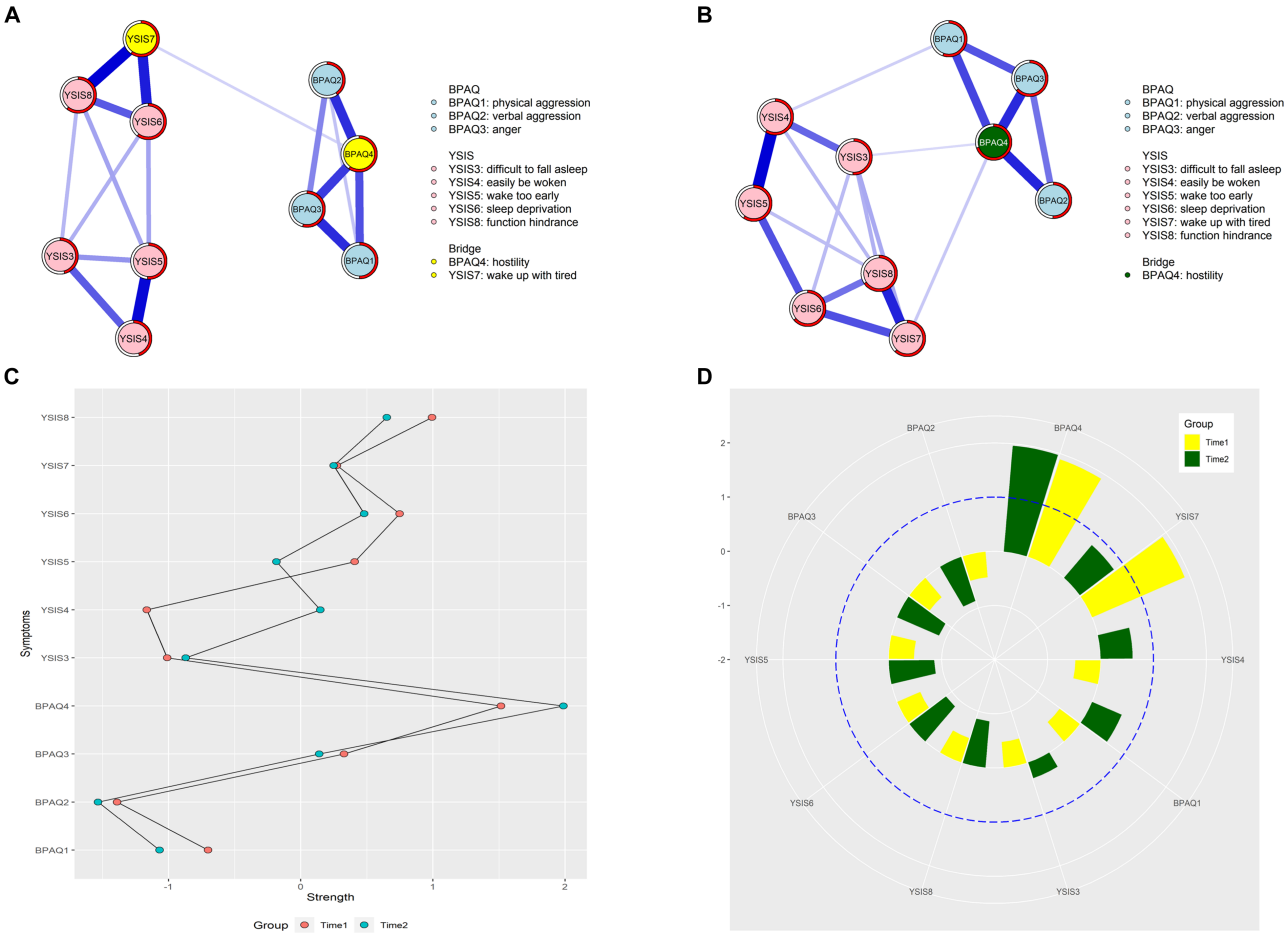
Network structures and centrality indexes. **(A)** The network structure at the first time point. **(B)** The network structure at the second time point. **(C)** The *strength* centrality values between two-time points. **(D)** The bridge centrality values between two-time points.

The aggression-sleep disturbance networks at two time points are shown in Figure 1 and Supplementary Figure S1, and weighted adjacency matrices are shown in Supplementary Tables S1, S2.

For the first time point, 17 edges were not zero (38%) among 45 possible edges, and all edges were positive. The edge of “wake up with tired” – “function hindrance” (YSIS7 – YSIS8) showed the strongest association, followed by the edge of “easily be woken” – “wake up too early” (YSIS4 – YSIS5) and the edge of “sleep deprivation” – “wake up with tired” (YSIS6 – YSIS7), see Figure 1A. For the second time point, 19 edges were not zero (42%) among 45 possible edges, and all edges were positive. The edge of “easily be woken” – “wake up too early” (YSIS4 – YSIS5) showed the strongest association, followed by the edge of “wake up with tired” – “function hindrance” (YSIS7 – YSIS8) and the edge of “verbal aggression” – “hostility” (BPAQ2-BPAQ4), see Figure 1B.

In Figure 1C, “hostility” (BPAQ4) had the highest node *EI*, followed by “function hindrance” (YSIS8) and “sleep deprivation” (YSIS6) at the first time point. Each node’s neighbors could potentially account for an average of 53% of the variance (*M*_predictability_ = 0.53 ± 0.07). Similarly, in Figure 1C, “hostility” (BPAQ4) had the highest node *EI*, followed by “function hindrance” (YSIS8) and “sleep deprivation” (YSIS6) at the second time point. Each node’s neighbors could potentially account for an average of 60% of the variance (*M*_predictability_ = 0.60 ± 0.06).

“Hostility” (BPAQ4) and “wake up with tired” (YSIS7) emerged as the bridge symptoms at the first time point (see Figure 1D). However, only “hostility” (BPAQ4) emerged as the bridge symptom at the second time point (see Figure 1D).

### 3.3 Cross-lagged panel network

**Figure 2:**
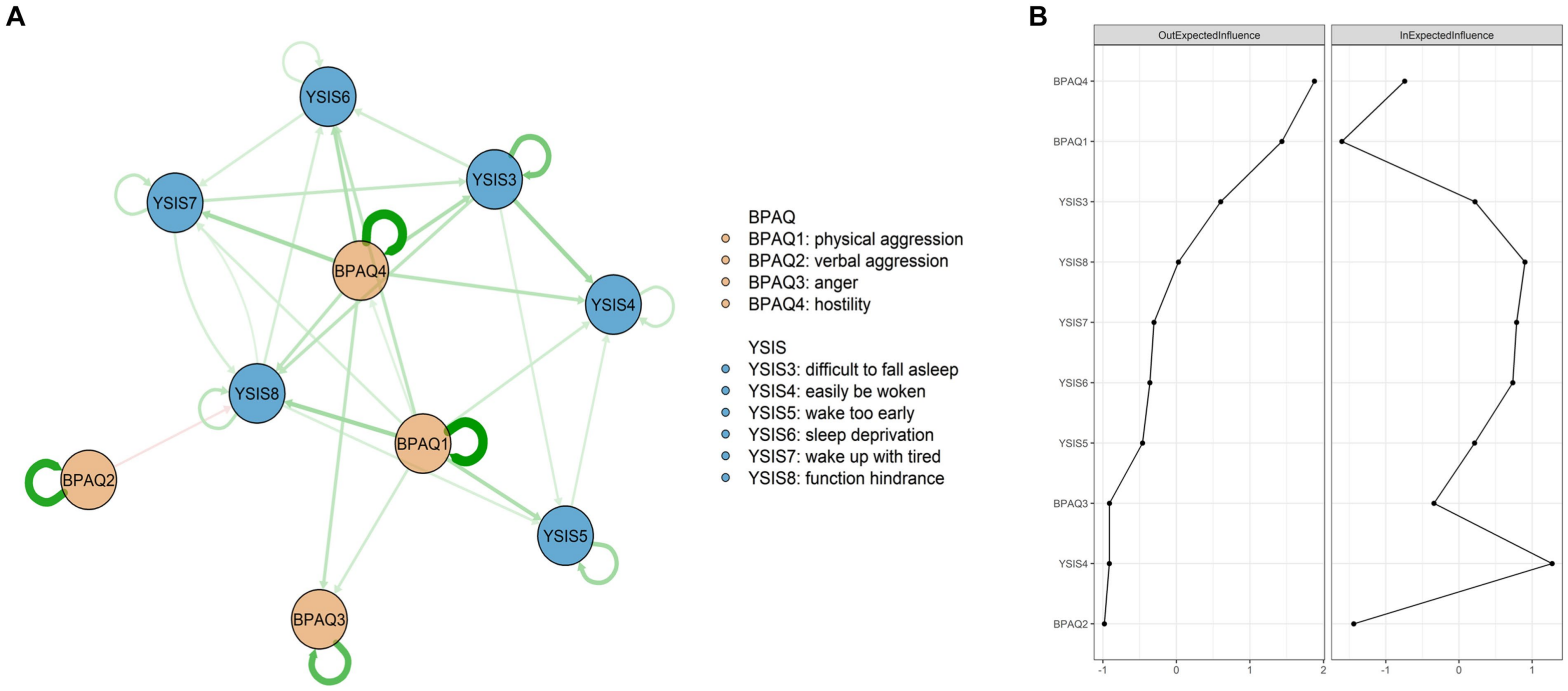
Network structures and centrality indexes. **(A)** The *OEI* and *IEI* values of all nodes. **(B)** The CLPN structure.

The CLPN structure is shown in Figure 2A, and all edge weights are shown in LASSO cross-lagged regression matrices in Supplementary Table S3.

A total of 68 edges were not zero (68%) among 100 possible edges. Except for autoregression paths, the edge of “difficulty in falling asleep” – “easily be woken” (YSIS3 – YSIS4) showed the strongest cross-lagged association, followed by the edge of “physical aggression” – “function hindrance” (BPAQ1 – YSIS8) and the edge of “hostility” – “wake up with tired” (BPAQ4 – YSIS7). Figure 2B shows the *OEI* and *IEI* values. “Hostility” (BPAQ4) had the highest node *OEI*, followed by “physical aggression” (BPAQ1) and “difficulty in falling asleep” (YSIS3). “Easily be woken” (YSIS4) had the highest node *IEI*, followed by “function hindrance” (YSIS8) and “wake up with tired” (YSIS7). Autoregression paths are shown in Supplementary Figure S2.

### 3.4 Network accuracy and stability

**Figure 3:**
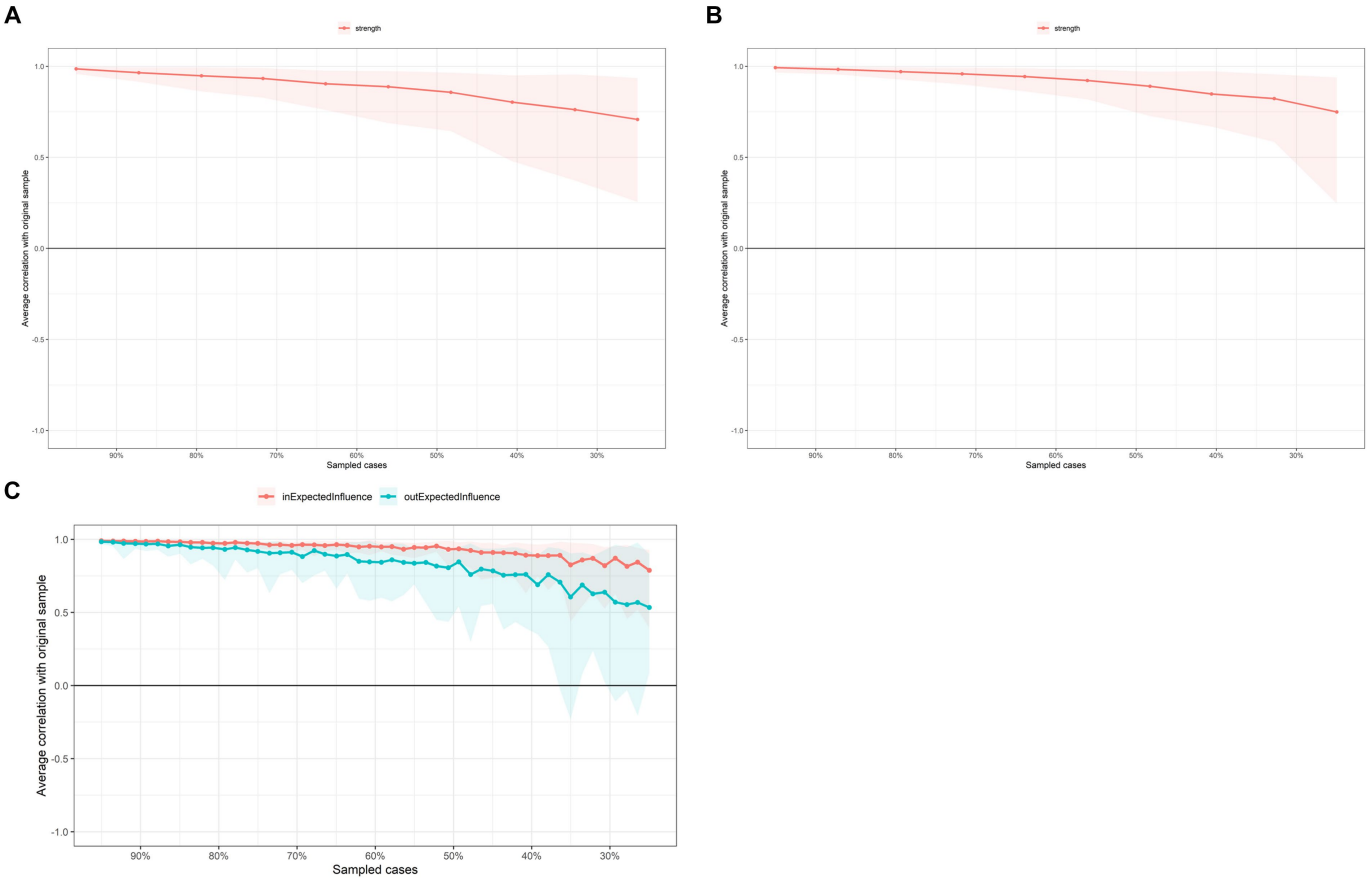
Case-dropping bootstrap test of centrality indices. The x-axis indicates the percentage of cases in the original sample included at each step. The y-axis indicates the correlations between the centrality indices from the original network and the indices from the networks re-estimated after excluding increasing percentages of cases. **(A)** First-time point. **(B)** Second time point. **(C)** CLPN.

In Figure 3, the case-dropping bootstrap procedure showed that *CS-Cs* of *EI* at the first and second time points were 0.44 and 0.59, respectively. The *CS-Cs* of *OEI* and *IEI* were 0.42 and 0.71, respectively. Case-dropping test results indicated good stability for centrality indicators. 95% of bootstrapped *CI*s of edges were narrow (Supplementary Figure S3), suggesting that edges were trustworthy. The results of the non-parametric bootstrap procedure revealed that most comparisons among edge weights and centrality indicators were statistically significant (Supplementary Figures S4–S6).

### 3.5 Network comparison between the baseline and the second time point

**Figure 4:**
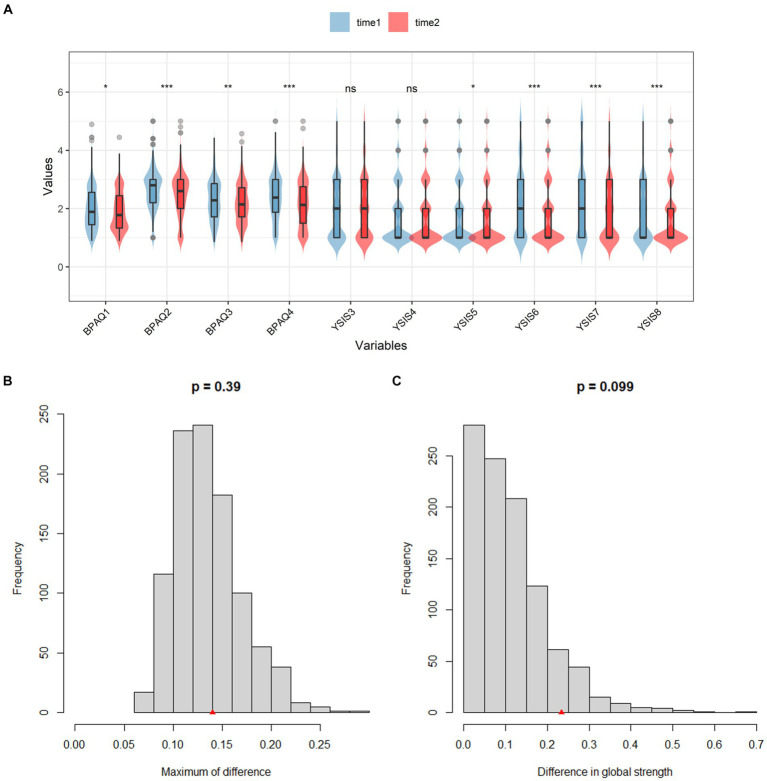
*T*-test results of all nodes and NCT results between two-time points. **(A)** The *t*-test results of BPAQ and YSIS. **(B)** The network edge invariance. **(C)** The network global invariance.

The *t*-test result is shown in Figure 4A, except for “*difficulty in falling asleep*” and “*easily be woken*,” other symptoms decreased significantly. NCT results are shown in Figure 4. The value of the maximum difference in any edge weights (1,000 permutations) was not significant (*M* = 0.14, *p* = 0.39) (Figure 4B). The value of the difference in global network strength was also not significant (baseline = 4.31; second time = 4.60, *p* = 0.10) (Figure 4C).

## 4 Discussion

To the best of our knowledge, this is the first study on sleep disturbance and aggression among college students during COVID-19. In the research, data were collected from 665 college students regarding their sleep quality and aggressive behaviors during the period of campus closure and post-closure. Two cross-sectional symptom networks and a CLPN were formed for further analysis. Several points in the study are worth discussing.

The most critical node in the networks of waves 1 and 2 was hostility, a psychological dimension of aggression known for its more detrimental impact on interpersonal relationships compared to physical aggression (62). Notably, hostility served as the connecting link between aggression and sleep disturbance in both waves, consistent with the findings of Sun et al. (63). The pandemic has the potential to induce significant psychological distress (64), often manifesting as hostility (30). Furthermore, our results align with a key discovery regarding the long-term effects of COVID-19: even after the lifting of containment measures, college students may continue to experience affective and somatic symptoms such as anxiety, sleep disturbance, and hostility, albeit to a lesser extent (65). Considering another factor, sleep disturbance, our findings echo the research conducted by Granö et al. (66) on employees, indicating a correlation between sleep disturbance and hostility across different populations. Bringing together all the relevant factors discussed in our study, it becomes evident that during the COVID-19 pandemic, societal factors contributed to increased engagement in online courses, internet usage, and video games, all of which were directly linked to sleep disturbance and, consequently, heightened hostility (67, 68).

In the two networks of waves 1 and 2, symptoms underlying sleep disturbance were significantly associated, particularly “easily be woken” – “wake up too early” and “wake up with tired” – “function hindrance.” On the one hand, this association highlights a mutually reinforcing relationship. For instance, a college student who tends to wake up too early is also more likely to be easily awakened and vice versa. Similarly, the connection between “wake up with tired” and “function hindrance” follows a similar pattern. On the other hand, our findings, consistent with the GAM, indicate that two associations between sleep disturbances can be attributed to biological factors, as supported by various studies (11). In summary, COVID-19 serves as a significant catalyst for sleep disturbance, further exacerbating aggressive behaviors (39, 69). Additionally, when individuals find themselves in aggressive environments, such as those prevalent on college campuses, they are more likely to experience sleep disturbances (70).

In addition to examining symptom networks, we investigated changes in networks during the closure period and after the lifting of closures. With the exception of difficulty in falling asleep and easily be woken, two symptoms of sleep disturbance did not show significant changes in statistics, all other symptoms decreased 1 month after the containment measures were lifted. Previous studies have yielded varied results, primarily due to differences in considering all dimensions of sleep disturbance. In our current study, we obtained similar results to those of Salfi et al. (41) in Italy, indicating that although most sleep disturbance symptoms diminish after home quarantine, COVID-19 still has a long-term effect on reducing the sleep quality of college students. Regarding aggression, our findings provide further insights from two perspectives. First, during the closure period, the uncertainties brought about by COVID-19 led to a greater increase in aggressive behavior than usual (21, 22, 71). Additionally, our results strongly support the GAM, suggesting that closure can serve as a triggering situation for aggressive decision-making. Consequently, when such circumstances dissipate, the corresponding aggressive behavior also significantly decreases.

At the disease level, sleep disturbance, as a comprehensive issue, can potentially lead to aggressive behaviors (72). However, as indicated by Bubier and Drabick (73), conclusions drawn at the symptom level may even overturn results obtained at the disease level. In our current study, physical aggression and hostility emerge as two symptoms capable of inducing other symptoms. In essence, while sleep disturbance may seem to cause aggressive behaviors when viewed broadly, a closer examination of symptoms reveals that aggressive symptoms can trigger other symptoms within the network. Physical aggression entails intentional actions aimed at physically harming others (74). In college students, physical aggression can be predicted by personality traits such as low agreeableness, high extraversion, and high conscientiousness (75). Additionally, anger is identified as a critical risk factor for physical aggression among college students (76). Observations by Ostrov et al. (77) suggest that, typically, college students resort to physical aggression when they find themselves victimized in peer relations, which can exacerbate hostile attribution bias. Drawing from our results and previous observations, and considering the GAM, in the context of the COVID-19 pandemic, college students experiencing rejection or low-quality relationships with peers may resort to physical aggression as a coping mechanism, particularly when they exhibit impulsive traits or other personality characteristics such as low extraversion.

In terms of hostility, our results successfully replicate the findings of Shapiro et al. (78), indicating that negative mood and hostility can elevate blood pressure during sleep. Moreover, our results suggest that, on the one hand, the isolation resulting from COVID-19 is also a risk factor for hostility among college students (79). On the other hand, our findings provide somatic evidence of the chain effect of COVID-19 on college students. During the closure period, students tend to increase their internet usage for online courses, and this heightened social isolation may predispose them to internet addiction or substance abuse (80). In our current study, we take a step further by showing that during the closure, college students who exhibit problematic internet use are more likely to display hostility both online and offline (81).

An important factor worth noting is waking up too early. This finding aligns with previous studies on the impact of COVID-19 on the sleep quality of college students (18, 82, 83). During closures, negative news, academic stress, depression, and interpersonal relationships can act as early risk factors (65, 84). Our results reveal an additional pathway: aggressive behaviors such as “physical aggression” and “hostility” can trigger early awakening.

## 5 Limitations

In the current study, we disclose features of sleep disturbance and aggressive behaviors via longitudinal data. However, several shortcomings should be mentioned for further research. First, in the current research, self-report scales are utilized as tools to measure the tendency toward aggression and sleep disturbance. In the future, researchers can incorporate additional methods to facilitate diagnosis. Second, the current generalizability of the results is somewhat limited due to the inadequate consideration of whether participants have been diagnosed with insomnia disorders in the past or present, as well as factors measuring participants’ levels of academic stress during participant recruitment. Finally, since aggression and sleep disturbance are closely related to depression and anxiety, future studies could include more variables to enable more precise speculation (81).

## 6 Conclusion

In the current study, with longitudinal data, we disclose the bidirectional relationship between sleep disturbance and aggression. In the cross-sectional symptom network, hostility is the critical symptom both in waves 1 and 2. Furthermore, hostility can cause aggression and sleep disturbance in college students. In a time sequence, difficulty in falling asleep and easily be woken did not change after closure lifted significantly, whereas other symptoms declined. Two symptoms, physical aggression and hostility, can trigger other symptoms and easily be woken, which are induced by other symptoms.

## Data availability statement

The raw data supporting the conclusions of this article will be made available by the authors, without undue reservation.

## Ethics statement

The studies involving humans were approved by the research was examined and approved by the Ethics Committee of the First Author's Affiliated Institution. The studies were conducted in accordance with the local legislation and institutional requirements. The participants provided their written informed consent to participate in this study.

## Author contributions

JZ: Formal analysis, Methodology, Writing – original draft. BB: Investigation, Writing – review & editing. ML: Conceptualization, Supervision, Writing – review & editing. GL: Conceptualization, Funding acquisition, Supervision, Writing – review & editing.

## Footnotes

^1^ https://www.wjx.cn

## References

[1] Bedford J Enria D Giesecke J Heymann DL Ihekweazu C Kobinger G. COVID-19: towards controlling of a pandemic. Lancet. (2020) 395:1015–8. doi: 10.1016/s0140-6736(20)30673-5, PMID: 32197103 PMC7270596

[2] Sahu P. Closure of universities due to coronavirus disease 2019 (COVID-19): Impact on Education and mental health of students and academic staff. Cureus. (2020) 12:e7541. doi: 10.7759/cureus.7541, PMID: 32377489 PMC7198094

[3] Deng J Zhou F Hou W Silver Z Wong CY Chang O. The prevalence of depressive symptoms, anxiety symptoms and sleep disturbance in higher education students during the COVID-19 pandemic: a systematic review and meta-analysis. Psychiatry Res. (2021) 301:113863. doi: 10.1016/j.psychres.2021.113863, PMID: 33984824 PMC9225824

[4] Ara T Rahman MM Hossain MA Ahmed A. Identifying the associated risk factors of sleep disturbance during the COVID-19 lockdown in Bangladesh: a web-based survey. Front Psych. (2020) 11:580268. doi: 10.3389/fpsyt.2020.580268, PMID: 33093839 PMC7527420

[5] Tao Y Tang Q Wang S Zou X Ma Z Zhang L. The impact of long-term online learning on social anxiety and problematic smartphone use symptoms among secondary school students with different levels of fear of missing out: evidence from a symptom network and longitudinal panel network analysis. J Behav Addict. (2024). doi: 10.1556/2006.2023.00081 [Epubh ahead of print]., PMID: 38206330 PMC10988399

[6] Tao Y Tang Q Zou X Wang S Ma Z Liu X. The impact of long-term online learning on internet addiction symptoms among depressed secondary school students: insights from a Cross-panel network analysis. Behav Sci (Basel). (2023) 13:520. doi: 10.3390/bs13070520, PMID: 37503967 PMC10376411

[7] Niu H Wang S Tao Y Tang Q Zhang L Liu X. The association between online learning, parents' marital status, and internet addiction among adolescents during the COVID-19 pandemic period: a cross-lagged panel network approach. J Affect Disord. (2023) 333:553–61. doi: 10.1016/j.jad.2023.04.096 37127119

[8] Randler C Vollmer C. Aggression in young adults--a matter of short sleep and social jetlag? Psychol Rep. (2013) 113:754–65. doi: 10.2466/16.02.PR0.113x31z7 24693810

[9] Kamphuis J Dijk DJ Spreen M Lancel M. The relation between poor sleep, impulsivity and aggression in forensic psychiatric patients. Physiol Behav. (2014) 123:168–73. doi: 10.1016/j.physbeh.2013.10.015, PMID: 24184508

[10] Gallegos AM Trabold N Cerulli C Pigeon WR. Sleep and interpersonal violence: a systematic review. Trauma Violence Abuse. (2021) 22:359–69. doi: 10.1177/1524838019852633 31131736

[11] Van Veen MM Karsten J Verkes R-J Lancel M. Sleep quality is associated with aggression in forensic psychiatric patients, independent of general psychopathology. J Forensic Psychiatry Psychol. (2020) 31:699–713. doi: 10.1080/14789949.2020.1785526

[12] van Staaden MJ Searcy WA Hanlon RT. Signaling aggression. Adv Genet. (2011) 75:23–49. doi: 10.1016/b978-0-12-380858-5.00008-3 22078476

[13] Allen JJ Anderson CA Bushman BJ. The general aggression model. Curr Opin Psychol. (2018) 19:75–80. doi: 10.1016/j.copsyc.2017.03.034 29279227

[14] Oliveira Carvalho P Hülsdünker T Carson F. The impact of the COVID-19 lockdown on European Students' negative emotional symptoms: a systematic review and Meta-analysis. Behav Sci (Basel). (2021) 12:3. doi: 10.3390/bs12010003, PMID: 35049614 PMC8772797

[15] Wei Z Hu Y Xiao J Wang R Huang Q Peng Z. Impacts of the psychological stress response on aggression in adolescents during the COVID-19 epidemic in China. J Pac Rim Psychol. (2022) 16:183449092211025. doi: 10.1177/18344909221102579

[16] Tao Y Niu H Li Y Liu X Wang S Ma Z. Effects of personal relative deprivation on the relationship between anger rumination and aggression during and after the COVID-19 pandemic lockdown: a longitudinal moderated network approach. J Adolesc. (2023) 95:596–608. doi: 10.1002/jad.12140, PMID: 36638841

[17] Spencer C Mallory AB Cafferky BM Kimmes JG Beck AR Stith SM. Mental health factors and intimate partner violence perpetration and victimization: a meta-analysis. Psychol Violence. (2019) 9:1–17. doi: 10.1037/vio0000156

[18] Tao Y Hou W Niu H Ma Z Zhang S Zhang L. Centrality and bridge symptoms of anxiety, depression, and sleep disturbance among college students during the COVID-19 pandemic-a network analysis. Curr Psychol. (2022):1–12. doi: 10.1007/s12144-022-03443-x [Epubh ahead of print]., PMID: 35967497 PMC9362556

[19] Tao Y Tang Q Zou X Wang S Ma Z Zhang L. Effects of attention to negative information on the bidirectional relationship between fear of missing out (FoMO), depression and smartphone addiction among secondary school students: evidence from a two-wave moderation network analysis. Comput Hum Behav. (2023) 148:107920. doi: 10.1016/j.chb.2023.107920

[20] Wang S Hou W Tao Y Ma Z Li K Wang Y. Mapping network connection among symptoms of anxiety, depression, and sleep disturbance in Chinese high school students. Front Public Health. (2022) 10:1015166. doi: 10.3389/fpubh.2022.1015166, PMID: 36466464 PMC9710521

[21] Mazza M Marano G Lai C Janiri L Sani G. Danger in danger: interpersonal violence during COVID-19 quarantine. Psychiatry Res. (2020) 289:113046. doi: 10.1016/j.psychres.2020.113046, PMID: 32387794 PMC7190494

[22] Overall NC Chang VT Cross EJ Low RST Henderson AME. Sexist attitudes predict family-based aggression during a COVID-19 lockdown. J Fam Psychol. (2021) 35:1043–52. doi: 10.1037/fam0000834, PMID: 33734757

[23] Birmingham WC Wadsworth LL Lassetter JH Graff TC Lauren E Hung M. COVID-19 lockdown: impact on college students' lives. J Am Coll Heal. (2023) 71:879–93. doi: 10.1080/07448481.2021.1909041 34292141

[24] Körmükçü FY. Research on the effects of the covid-19 quarantine process on the aggression levels of university students. Pak J Med Health Sci. (2021) 15:2256–60. doi: 10.53350/pjmhs211572256

[25] Fung ALC. The significance of family structure in internalizing (anxious/depressed) and externalizing (aggressive/delinquent) problems among Chinese adolescents. Appl Res Qual Life. (2021) 16:2403–18. doi: 10.1007/s11482-021-09923-9

[26] George EM Rosvall KA. Bidirectional relationships between testosterone and aggression: a critical analysis of four predictions. Integr Comp Biol. (2022) 62:474–86. doi: 10.1093/icb/icac100 35759399 PMC9494517

[27] Murray-Close D Crick NR Tseng WL Lafko N Burrows C Pitula C. Physiological stress reactivity and physical and relational aggression: the moderating roles of victimization, type of stressor, and child gender. Dev Psychopathol. (2014) 26:589–603. doi: 10.1017/s095457941400025x, PMID: 25047286

[28] Fraser AM Stockdale LA Bryce CI Alexander BL. College students’ media habits, concern for themselves and others, and mental health in the era of COVID-19. Psychol Pop Media. (2022) 11:139–51. doi: 10.1037/ppm0000345

[29] Bessière K Kiesler S Kraut R Boneva BS. Effects of internet use and social resources on changes in depression. Inf Commun Soc. (2008) 11:47–70. doi: 10.1080/13691180701858851

[30] Barlett CP Anderson CA. Bad news, bad times, and violence: the link between economic distress and aggression. Psychol Violence. (2014) 4:309–21. doi: 10.1037/a0034479

[31] Dubar RT Watkins NK Hill GC. Examining the direction of effects between COVID-19 experiences, general well-being, social media engagement, and insomnia symptoms among university students. Emerg Adulthood. (2021) 9:655–69. doi: 10.1177/21676968211051161

[32] Bronsard G Bartolomei F. Rhythms, rhythmicity and aggression. J Physiol Paris. (2013) 107:327–34. doi: 10.1016/j.jphysparis.2013.03.002 23542545

[33] Mogavero F Jager A Glennon JC. Clock genes, ADHD and aggression. Neurosci Biobehav Rev. (2018) 91:51–68. doi: 10.1016/j.neubiorev.2016.11.002, PMID: 27836462

[34] Tasso AF Hisli Sahin N San Roman GJ. COVID-19 disruption on college students: academic and socioemotional implications. Psychol Trauma. (2021) 13:9–15. doi: 10.1037/tra0000996, PMID: 33382329

[35] Bersani FS Barchielli B Ferracuti S Panno A Carbone GA Massullo C. The association of problematic use of social media and online videogames with aggression is mediated by insomnia severity: a cross-sectional study in a sample of 18- to 24-year-old individuals. Aggress Behav. (2022) 48:348–55. doi: 10.1002/ab.22008, PMID: 34870339

[36] Adachi PJ Willoughby T. Demolishing the competition: the longitudinal link between competitive video games, competitive gambling, and aggression. J Youth Adolesc. (2013) 42:1090–104. doi: 10.1007/s10964-013-9952-2, PMID: 23595418

[37] Young R Len-Ríos M Young H. Romantic motivations for social media use, social comparison, and online aggression among adolescents. Comput Hum Behav. (2017) 75:385–95. doi: 10.1016/j.chb.2017.04.021

[38] Serpeloni F Radtke KM Hecker T Sill J Vukojevic V de Assis SG. Does prenatal stress shape postnatal resilience? – an epigenome-wide study on violence and mental health in humans. Front Genet. (2019) 10:269. doi: 10.3389/fgene.2019.00269, PMID: 31040859 PMC6477038

[39] Lindberg N Tani P Appelberg B Naukkarinen H Rimón R Porkka-Heiskanen T. Human impulsive aggression: a sleep research perspective. J Psychiatr Res. (2003) 37:313–24. doi: 10.1016/s0022-3956(03)00041-4, PMID: 12765854

[40] Paiva T Canas-Simião H. Sleep and violence perpetration: a review of biological and environmental substrates. J Sleep Res. (2022) 31:e13547. doi: 10.1111/jsr.13547, PMID: 35037316

[41] Salfi F Amicucci G Corigliano D Viselli L D'Atri A Tempesta D. Two years after lockdown: longitudinal trajectories of sleep disturbances and mental health over the COVID-19 pandemic, and the effects of age, gender and chronotype. J Sleep Res. (2023) 32:e13767. doi: 10.1111/jsr.13767, PMID: 36317491 PMC9878065

[42] Buss AH Perry M. The Aggression Questionnaire. J Pers Soc Psychol. (1992) 63:452–9. doi: 10.1037/0022-3514.63.3.452 1403624

[43] Buysse DJ Ancoli-Israel S Edinger JD Lichstein KL Morin CM. Recommendations for a standard research assessment of insomnia. Sleep. (2006) 29:1155–73. doi: 10.1093/sleep/29.9.1155 17040003

[44] Marchetti I. Hopelessness: a network analysis. Cogn Ther Res. (2019) 43:611–9. doi: 10.1007/s10608-018-9981-y

[45] Borsboom D Cramer AOJ. Network analysis: an integrative approach to the structure of psychopathology. Annu Rev Clin Psychol. (2013) 9:91–121. doi: 10.1146/annurev-clinpsy-050212-185608 23537483

[46] Epskamp S Borsboom D Fried EI. Estimating psychological networks and their accuracy: a tutorial paper. Behav Res Methods. (2018) 50:195–212. doi: 10.3758/s13428-017-0862-1, PMID: 28342071 PMC5809547

[47] Jones PJ Ma R McNally RJ. Bridge centrality: a network approach to understanding comorbidity. Multivariate Behav Res. (2021) 56:353–67. doi: 10.1080/00273171.2019.1614898, PMID: 31179765

[48] Li W Zhang Q Tang Y Park SC Park Y Yang SY. Network analysis of psychiatric symptoms in schizophrenia: findings from the research on Asian psychotropic prescription patterns for antipsychotics (REAP-AP). Asian J Psychiatr. (2022) 75:103200. doi: 10.1016/j.ajp.2022.103200, PMID: 35850062

[49] Hirota T Deserno M McElroy E. The network structure of irritability and aggression in individuals with autism Spectrum disorder. J Autism Dev Disord. (2020) 50:1210–20. doi: 10.1007/s10803-019-04354-w, PMID: 31897854

[50] Kilius E Abbas NH McKinnon L Samson DR. Pandemic nightmares: COVID-19 lockdown associated with increased aggression in female university Students' dreams. Front Psychol. (2021) 12:644636. doi: 10.3389/fpsyg.2021.644636, PMID: 33746860 PMC7973031

[51] Maxwell JP. Development and preliminary validation of a Chinese version of the Buss-Perry aggression questionnaire in a population of Hong Kong Chinese. J Pers Assess. (2007) 88:284–94. doi: 10.1080/00223890701317004, PMID: 17518550

[52] Liu X Yang Y Liu Z-Z Luo Y Fan F Jia C. Psychometric properties of youth self-rating insomnia scale (YSIS) in Chinese adolescents. Sleep Biol Rhythms. (2019) 17:339–48. doi: 10.1007/s41105-019-00222-3 PMC10900025 38476857

[53] R Core Team. A language and environment for statistical computing. (2022). Available at: https://www.R-project.org/.

[54] Jones P. Networktools: tools for identifying important nodes in networks (1.5.0). (2022). Available at: https://CRAN.R-project.org/package=networktools.

[55] Epskamp S Fried EI. A tutorial on regularized partial correlation networks. Psychol Methods. (2018) 23:617–34. doi: 10.1037/met0000167, PMID: 29595293

[56] Epskamp S Cramer AO Waldorp LJ Schmittmann VD Borsboom D. Qgraph: network visualizations of relationships in psychometric data. J Stat Softw. (2012) 48:1–18. doi: 10.18637/jss.v048.i04

[57] Haslbeck JMB Waldorp LJ. Mgm: estimating time-varying mixed graphical models in high-dimensional data. J Stat Softw. (2020) 93:1–46. doi: 10.18637/jss.v093.i08

[58] Sánchez Hernández MO Carrasco MA Holgado-Tello FP. Anxiety and depression symptoms in Spanish children and adolescents: an exploration of comorbidity from the network perspective. Child Psychiatry Hum Dev. (2023) 54:736–49. doi: 10.1007/s10578-021-01286-4, PMID: 34797464 PMC10140092

[59] Tao Y Zou X Tang Q Hou W Wang S Ma Z. Mapping network connection and direction between anxiety and depression symptoms across the early, middle, and late adolescents: insights from a large Chinese sample. J Psychiatr Res. (2024) 169:174–83. doi: 10.1016/j.jpsychires.2023.11.035, PMID: 38039692

[60] Friedman J Hastie T Tibshirani R. Regularization paths for generalized linear models via coordinate descent. J Stat Softw. (2010) 33:1–22. doi: 10.18637/jss.v033.i01 20808728 PMC2929880

[61] van Borkulo CD van Bork R Boschloo L Kossakowski JJ Tio P Schoevers RA. Comparing network structures on three aspects: a permutation test. Psychol Methods. (2023) 28:1273–85. doi: 10.1037/met0000476, PMID: 35404628

[62] Sotskova A Woodin EM Gou LH. Hostility, flooding, and relationship satisfaction: predicting trajectories of psychological aggression across the transition to parenthood. Aggress Behav. (2015) 41:134–48. doi: 10.1002/ab.21570, PMID: 27539934

[63] Sun Y Wang L Li C Luo W. Sleep disturbance in Chinese college students with mental health problems: a moderated mediation model. Int J Environ Res Public Health. (2022) 19:19. doi: 10.3390/ijerph192114570, PMID: 36361449 PMC9653838

[64] Vitalakumar D Sharma A Kumar A Flora SJS. Neurological manifestations in COVID-19 patients: a meta-analysis. ACS Chem Neurosci. (2021) 12:2776–97. doi: 10.1021/acschemneuro.1c00353 34260855

[65] Wang Z Jiang B Wang X Niu Y Xue H. Cross-sectional investigation and correlation analysis of psychology of college students returning to campus after COVID-19 lockdown lift. Front Psych. (2022) 13:915042. doi: 10.3389/fpsyt.2022.915042, PMID: 35935405 PMC9352858

[66] Grano N Vahtera J Virtanen M Keltikangas-Jarvinen L Kivimaki M. Association of hostility with sleep duration and sleep disturbances in an employee population. Int J Behav Med. (2008) 15:73–80. doi: 10.1080/10705500801929510, PMID: 18569125

[67] Lam LT. Internet gaming addiction, problematic use of the internet, and sleep problems: a systematic review. Curr Psychiatry Rep. (2014) 16:444. doi: 10.1007/s11920-014-0444-1, PMID: 24619594

[68] Marciano L Ostroumova M Schulz PJ Camerini AL. Digital media use and Adolescents' mental health during the Covid-19 pandemic: a systematic review and Meta-analysis. Front Public Health. (2021) 9:793868. doi: 10.3389/fpubh.2021.793868, PMID: 35186872 PMC8848548

[69] Zhou SJ Wang LL Yang R Yang XJ Zhang LG Guo ZC. Sleep problems among Chinese adolescents and young adults during the coronavirus-2019 pandemic. Sleep Med. (2020) 74:39–47. doi: 10.1016/j.sleep.2020.06.001, PMID: 32836185 PMC7274988

[70] Brindle RC Cribbet MR Samuelsson LB Gao C Frank E Krafty RT. The relationship between childhood trauma and poor sleep health in adulthood. Psychosom Med. (2018) 80:200–7. doi: 10.1097/psy.0000000000000542, PMID: 29215455 PMC5794533

[71] Tao Y Niu H Hou W Zhang L Ying R. Hopelessness during and after the COVID-19 pandemic lockdown among Chinese college students: a longitudinal network analysis. J Clin Psychol. (2023) 79:748–61. doi: 10.1002/jclp.23439, PMID: 36037244 PMC9537977

[72] Kamphuis J Meerlo P Koolhaas JM Lancel M. Poor sleep as a potential causal factor in aggression and violence. Sleep Med. (2012) 13:327–34. doi: 10.1016/j.sleep.2011.12.006, PMID: 22305407

[73] Bubier JL Drabick DA. Co-occurring anxiety and disruptive behavior disorders: the roles of anxious symptoms, reactive aggression, and shared risk processes. Clin Psychol Rev. (2009) 29:658–69. doi: 10.1016/j.cpr.2009.08.005, PMID: 19729235 PMC2758916

[74] Thomas R. College student peer aggression: a review with applications for colleges and universities. Aggress Violent Behav. (2019) 48:218–29. doi: 10.1016/j.avb.2019.08.013

[75] Burton LA Hafetz J Henninger D. Gender differences in relational and physical aggression. Soc Behav Pers. (2007) 35:41–50. doi: 10.2224/sbp.2007.35.1.41

[76] Chen P Coccaro EF Jacobson KC. Hostile attributional bias, negative emotional responding, and aggression in adults: moderating effects of gender and impulsivity. Aggress Behav. (2012) 38:47–63. doi: 10.1002/ab.21407, PMID: 24833604 PMC4243523

[77] Ostrov JM Hart EJ Kamper KE Godleski SA. Relational aggression in women during emerging adulthood: a social process model. Behav Sci Law. (2011) 29:695–710. doi: 10.1002/bsl.1002, PMID: 21815200

[78] Shapiro D Jamner LD Goldstein IB. Daily mood states and ambulatory blood pressure. Psychophysiology. (1997) 34:399–405. doi: 10.1111/j.1469-8986.1997.tb02383.x 9260492

[79] Siegler IC Costa PT Brummett BH Helms MJ Barefoot JC Williams RB. Patterns of change in hostility from college to midlife in the UNC alumni heart study predict high-risk status. Psychosom Med. (2003) 65:738–45. doi: 10.1097/01.PSY.0000088583.25140.9C, PMID: 14508014

[80] Sun Y Li Y Bao Y Meng S Sun Y Schumann G. Brief report: increased addictive internet and substance use behavior during the COVID-19 pandemic in China. Am J Addict. (2020) 29:268–70. doi: 10.1111/ajad.13066, PMID: 32500608 PMC7300868

[81] Yen J-Y Yen C-F Wu H-Y Huang C-J Ko C-H. Hostility in the real world and online: the effect of internet addiction, depression, and online activity. Cyberpsychol Behav Soc Netw. (2011) 14:649–55. doi: 10.1089/cyber.2010.0393, PMID: 21476897

[82] Fatima Y Doi SA Mamun AA. Sleep quality and obesity in young subjects: a meta-analysis. Obes Rev. (2016) 17:1154–66. doi: 10.1111/obr.12444, PMID: 27417913

[83] Tang W Hu T Hu B Jin C Wang G Xie C. Prevalence and correlates of PTSD and depressive symptoms one month after the outbreak of the COVID-19 epidemic in a sample of home-quarantined Chinese university students. J Affect Disord. (2020) 274:1–7. doi: 10.1016/j.jad.2020.05.009, PMID: 32405111 PMC7217769

[84] Wu R Wang W Li W Zhao M Dewaele A Zhang WH. Sexual orientation and sleep problem among Chinese college students: mediating roles of interpersonal problems and depressive symptoms. J Affect Disord. (2021) 295:569–77. doi: 10.1016/j.jad.2021.08.075, PMID: 34509072

