# Supplementary material for: The interrelationship between sleep disturbance symptoms and aggression before and after the campus closure of the COVID-19 pandemic: insight from a cross-lagged panel network model
Source: Front Public Health. 2024 Mar 21;12:1357018. doi: 10.3389/fpubh.2024.1357018 (PMC10991807; doi:10.3389/fpubh.2024.1357018)

**Consent**

Dear students,

This is a psychological health tracking survey initiated by the Affiliated Brain Hospital of Nanjing Medical University during the period of isolation for college students on campus. The purpose of the survey is to understand the psychological status of college student groups and provide policy basis for scientific research and relevant departments. Our survey results are only used as scientific research and policy basis, and will not disclose participants' privacy and data results. In order to respect your privacy and match the data, this survey does not require you to fill in your class name, only your student ID truthfully, and answer according to the actual situation. Thank you for your participation, and we wish you a happy life and progress in your studies!

If you need psychological assistance, you can contact the Ministry of Education's psychological assistance hotline at 400-967-8920 (24 hours) at Central China Normal University or the free psychological counseling service at Beijing Normal University's Psychological Health Service Center at 010-86409146.

**Table S1.** The weighted adjacency matrix of BPAQ and YSIS symptoms (first time).

|  | BPAQ1 | BPAQ2 | BPAQ3 | BPAQ4 | YSIS3 | YSIS4 | YSIS5 | YSIS6 | YSIS7 | YSIS8 |
| --- | --- | --- | --- | --- | --- | --- | --- | --- | --- | --- |
| BPAQ1 | 0 | 0.097 | 0.340 | 0.280 | 0 | 0 | 0 | 0 | 0 | 0 |
| BPAQ2 | 0.097 | 0 | 0.200 | 0.340 | 0 | 0 | 0 | 0 | 0 | 0 |
| BPAQ3 | 0.340 | 0.200 | 0 | 0.320 | 0 | 0 | 0 | 0 | 0 | 0 |
| BPAQ4 | 0.280 | 0.340 | 0.320 | 0 | 0 | 0 | 0 | 0 | 0.072 | 0 |
| YSIS3 | 0 | 0 | 0 | 0 | 0 | 0.260 | 0.170 | 0.130 | 0 | 0.120 |
| YSIS4 | 0 | 0 | 0 | 0 | 0.260 | 0 | 0.400 | 0 | 0 | 0 |
| YSIS5 | 0 | 0 | 0 | 0 | 0.170 | 0.400 | 0 | 0.150 | 0 | 0.150 |
| YSIS6 | 0 | 0 | 0 | 0 | 0.130 | 0 | 0.150 | 0 | 0.360 | 0.260 |
| YSIS7 | 0 | 0 | 0 | 0.072 | 0 | 0 | 0 | 0.360 | 0 | 0.410 |
| YSIS8 | 0 | 0 | 0 | 0 | 0.120 | 0 | 0.150 | 0.260 | 0.410 | 0 |

**Table S2.** The weighted adjacency matrix of BPAQ and YSIS symptoms (second time).

|  | BPAQ1 | BPAQ2 | BPAQ3 | BPAQ4 | YSIS3 | YSIS4 | YSIS5 | YSIS6 | YSIS7 | YSIS8 |
| --- | --- | --- | --- | --- | --- | --- | --- | --- | --- | --- |
| BPAQ1 | 0 | 0 | 0.300 | 0.320 | 0 | 0.083 | 0 | 0 | 0 | 0 |
| BPAQ2 | 0 | 0 | 0.240 | 0.380 | 0 | 0 | 0 | 0 | 0 | 0 |
| BPAQ3 | 0.300 | 0.240 | 0 | 0.370 | 0 | 0 | 0 | 0 | 0 | 0 |
| BPAQ4 | 0.320 | 0.380 | 0.370 | 0 | 0.066 | 0 | 0 | 0 | 0.094 | 0 |
| YSIS3 | 0 | 0 | 0 | 0.066 | 0 | 0.270 | 0 | 0.120 | 0.150 | 0.140 |
| YSIS4 | 0.083 | 0 | 0 | 0 | 0.270 | 0 | 0.440 | 0 | 0 | 0.120 |
| YSIS5 | 0 | 0 | 0 | 0 | 0 | 0.440 | 0 | 0.300 | 0 | 0.110 |
| YSIS6 | 0 | 0 | 0 | 0 | 0.120 | 0 | 0.300 | 0 | 0.300 | 0.250 |
| YSIS7 | 0 | 0 | 0 | 0.094 | 0.150 | 0 | 0 | 0.300 | 0 | 0.380 |
| YSIS8 | 0 | 0 | 0 | 0 | 0.140 | 0.120 | 0.110 | 0.250 | 0.380 | 0 |

**Table S3.** LASSO cross-lagged regression matrix. Each number in the matrix represents the regression coefficient of the symptom in the same row on its left side (wave 1) predicting the symptom in the same column on its upper side (wave 2).

|  | BPAQ1 | BPAQ2 | BPAQ3 | BPAQ4 | YSIS3 | YSIS4 | YSIS5 | YSIS6 | YSIS7 | YSIS8 |
| --- | --- | --- | --- | --- | --- | --- | --- | --- | --- | --- |
| BPAQ1 | 0.46 | 0.00 | 0.08 | 0.06 | 0.03 | 0.08 | 0.14 | 0.11 | 0.07 | 0.16 |
| BPAQ2 | 0.00 | 0.39 | 0.02 | 0.03 | 0.00 | 0.00 | 0.00 | 0.00 | 0.00 | -0.05 |
| BPAQ3 | 0.00 | 0.00 | 0.30 | 0.00 | 0.00 | 0.00 | 0.00 | 0.00 | 0.01 | 0.00 |
| BPAQ4 | 0.00 | 0.05 | 0.11 | 0.44 | 0.14 | 0.12 | 0.04 | 0.14 | 0.15 | 0.12 |
| YSIS3 | 0.01 | 0.00 | 0.00 | 0.00 | 0.24 | 0.17 | 0.07 | 0.08 | 0.05 | 0.12 |
| YSIS4 | 0.00 | 0.00 | 0.00 | 0.01 | 0.00 | 0.10 | 0.01 | 0.00 | 0.00 | 0.00 |
| YSIS5 | 0.00 | 0.01 | 0.00 | 0.02 | 0.03 | 0.07 | 0.17 | 0.00 | 0.01 | 0.02 |
| YSIS6 | 0.02 | 0.01 | 0.02 | 0.03 | 0.00 | 0.03 | 0.00 | 0.08 | 0.07 | 0.00 |
| YSIS7 | 0.00 | 0.01 | 0.01 | 0.01 | 0.10 | 0.00 | 0.00 | 0.00 | 0.11 | 0.07 |
| YSIS8 | 0.03 | 0.00 | 0.01 | 0.02 | 0.03 | 0.02 | 0.07 | 0.07 | 0.05 | 0.12 |

**Figure S1.** The original network structures. A, time 1. B,
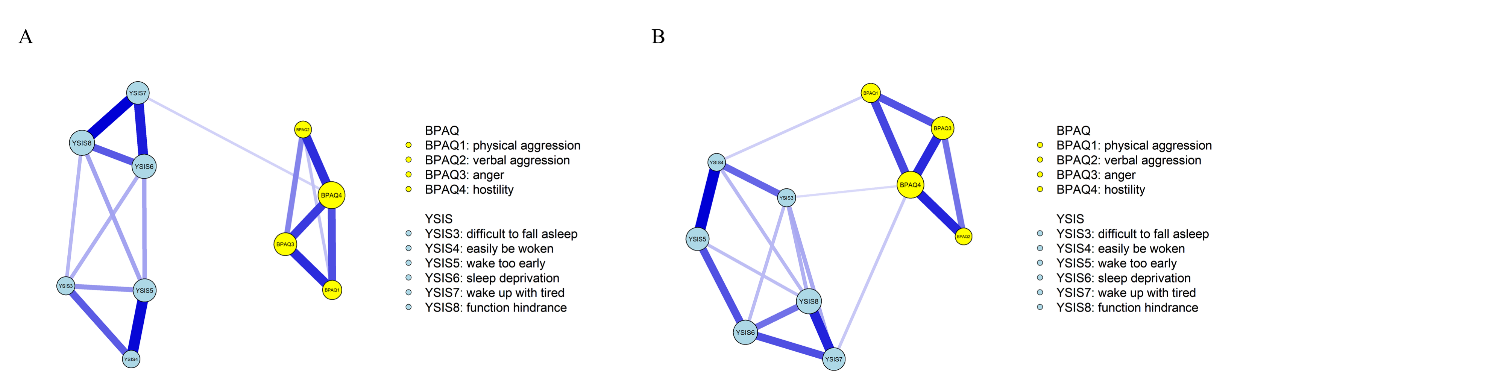
time 2.


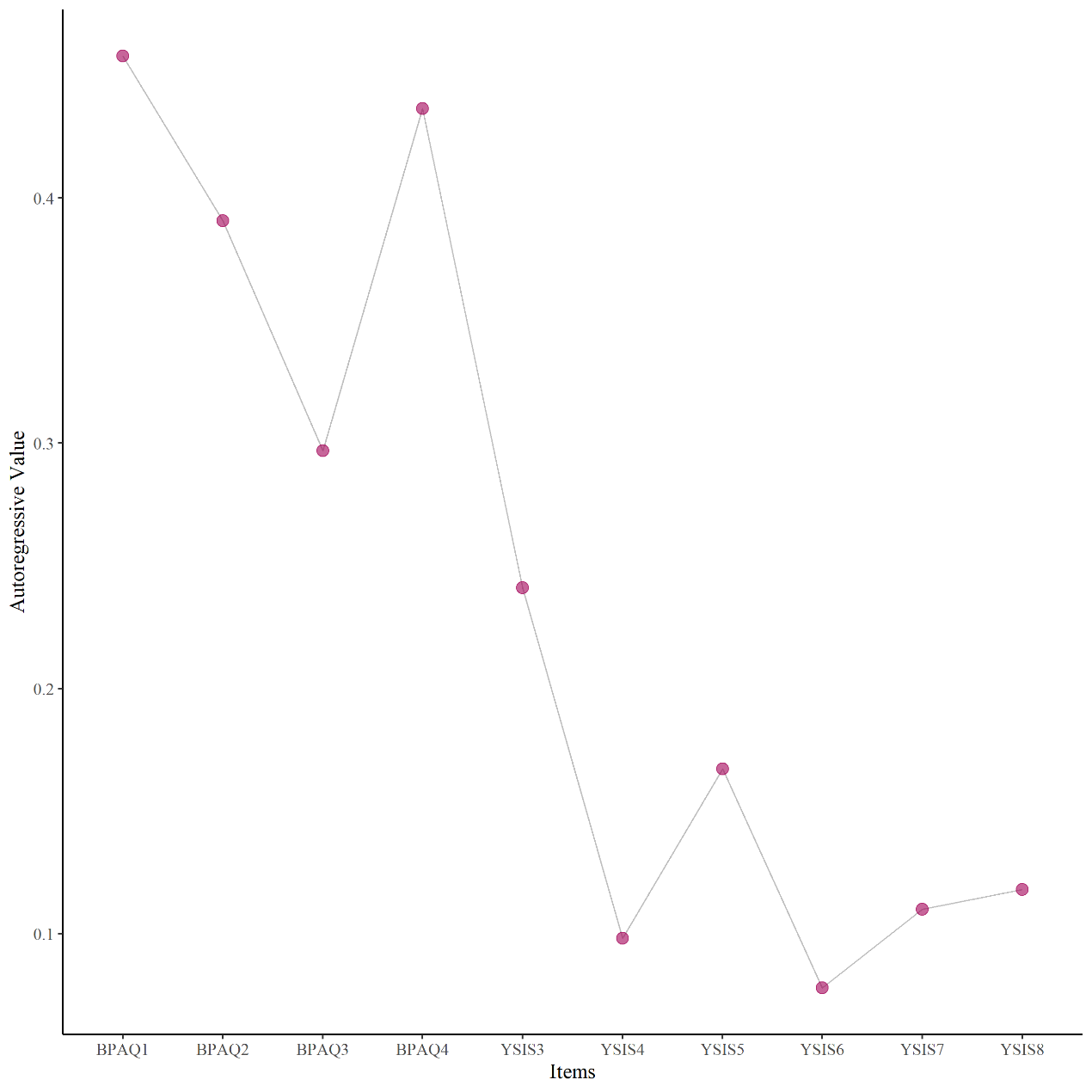


**Figure S2.** Autoregressive coefficients for each node.


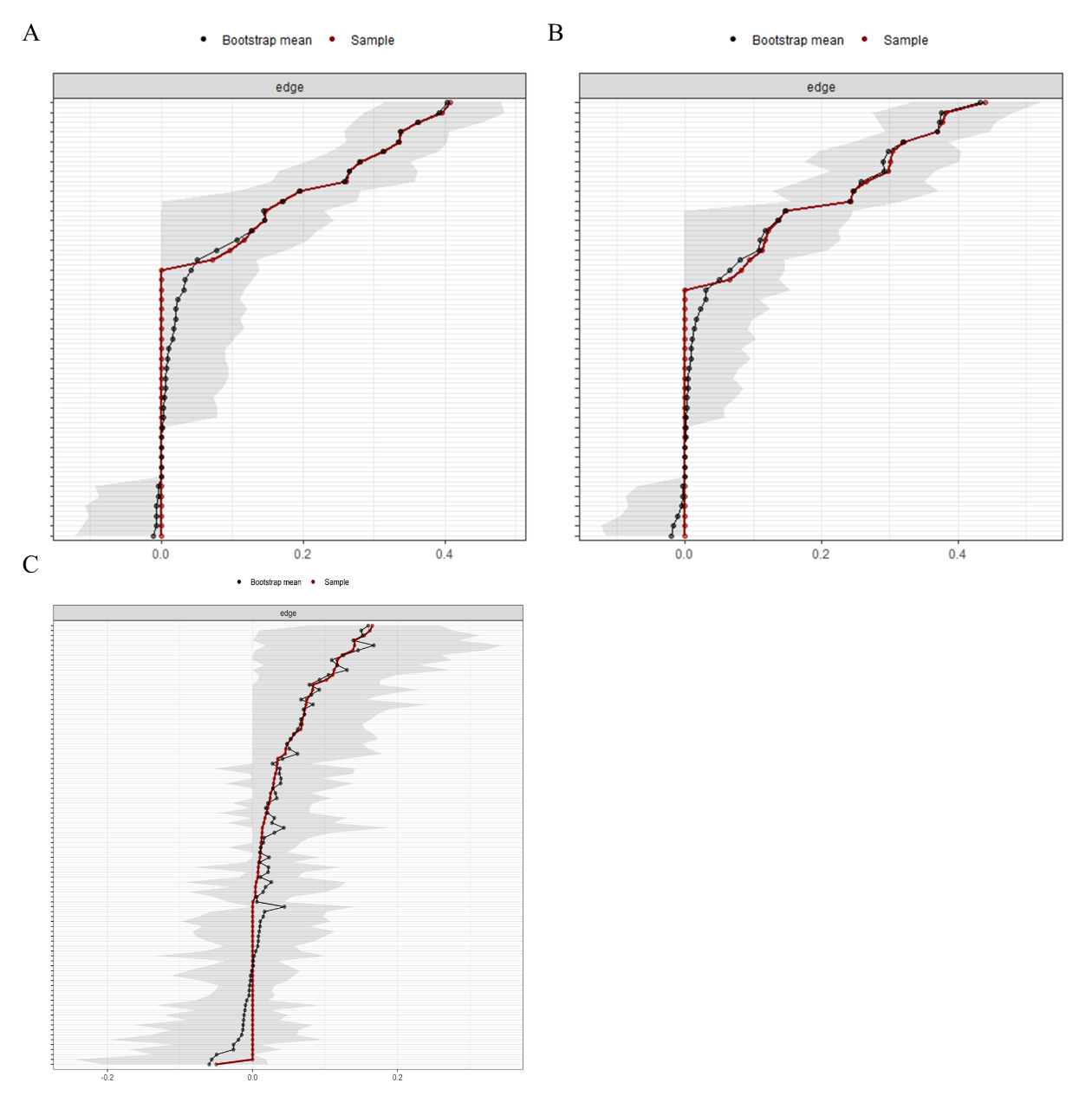
**Figure S3.** Nonparametric bootstrapped confidence intervals of estimated edges. The

red line represents the estimated edge, while the dark area indicates the 95% bootstrap

confidence interval. A, network at the first time point. B, network at the second time point. C, the CLPN.

**Figure S4.** The results of the bootstrapped difference tests (*α* = 0.05) for edge-weights were shown in this figure. The color of the boxes indicates whether edge-weights differ significantly from each other (i.e., black) or do not differ significantly (i.e., grey). A, network at the first time point. B, network at the second time point. C, the CLPN
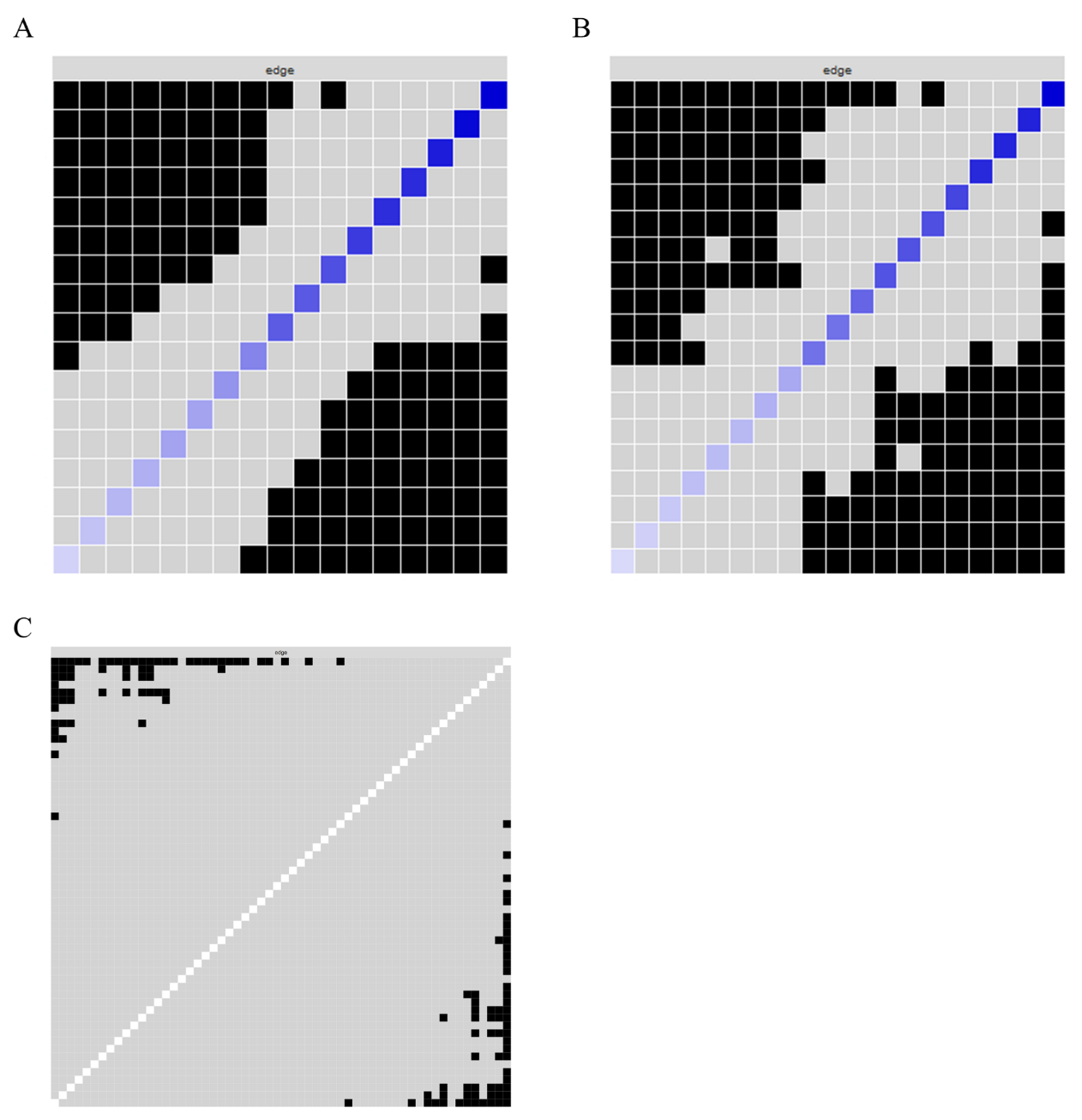
.


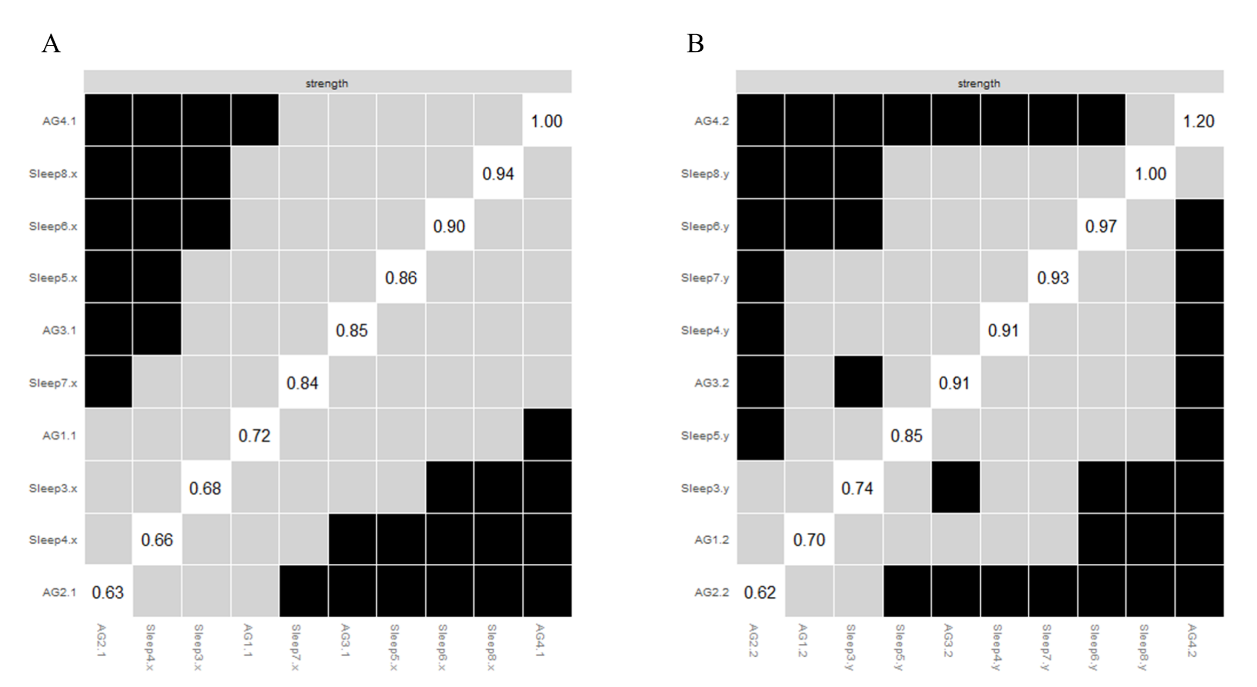
**Figure S5.** Nonparametric bootstrapped difference test for each node’s centrality strength. Grey boxes indicate no significant difference, whereas black boxes indicate a statistically significant difference (*p* < 0.05). A, network at the first time point. B, network at the second time point.

**Figure S6.** Nonparametric bootstrapped difference test for each node’s *OEI* and *IEI*. Grey boxes indicate no significant difference, whereas black boxes indicate a statistically significant difference (*p* < 0.05). A, *OEI*. B, *IEI*.
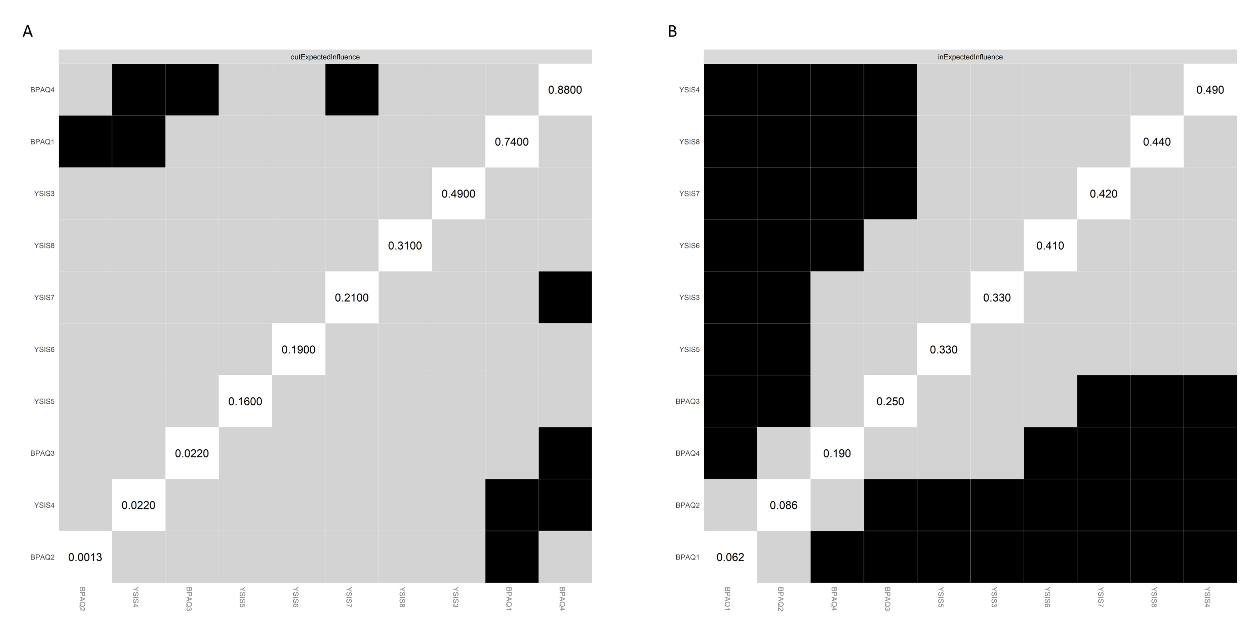

Supplement: Supplementary file 1 [file Data_Sheet_1.docx]
